# 5 kHz Transcranial Alternating Current Stimulation: Lack of Cortical Excitability Changes When Grouped in a Theta Burst Pattern

**DOI:** 10.3389/fnhum.2016.00683

**Published:** 2017-01-10

**Authors:** Patrik Kunz, Andrea Antal, Manuel Hewitt, Andreas Neef, Alexander Opitz, Walter Paulus

**Affiliations:** ^1^Department of Clinical Neurophysiology, University Medical Center GöttingenGöttingen, Germany; ^2^Department of Nonlinear Dynamics and Network Dynamics Group, Max Planck Institute for Dynamics and Self-OrganizationGöttingen, Germany; ^3^Nathan Kline Institute for Psychiatric ResearchOrangeburg, NY, USA

**Keywords:** transcranial alternating current stimulation, high frequency stimulation, theta burst stimulation, motor cortex, high intensity, safety

## Abstract

**Background**: Suprathreshold transcranial single pulse electrical stimulation (tES) is painful and not applicable in a repetitive mode to induce plastic after-effects.

**Objective**: In order to circumvent this pain problem, we applied here a 5 kHz transcranial alternating current stimulation (tACS) theta burst protocol with a field intensity of up to 10 mA to the primary motor cortex (M1). Furthermore, we were interested in finding out whether electrical theta burst stimulation (eTBS) is able to induce lasting after-effects on cortical plasticity.

**Methods**: Three different eTBS protocols were applied at 5 mA in a sham controlled, double blinded cross-over design on the M1 region of seventeen healthy subjects during the first part of the study. The second study part consists of three different eTBS protocols ranging from 5 mA to 10 mA and 1 ms to 5 ms sinusoidal bursts, applied to the M1 region of 14 healthy subjects.

**Results**: We were able to apply all eTBS protocols in a safe manner, with only six subjects reporting mild side effects related to the stimulation. However, no eTBS protocol induced lasting effects on muscle- evoked potential (MEP) amplitudes when compared to sham stimulation. Significant inhibition of MEP amplitude was only seen in the lower intensity protocols as compared to baseline.

**Conclusion**: eTBS is a safe method to apply high frequency tACS with up to 10 mA intensity. Future studies need to explore the parameter space to a larger extent in order to assure efficacy.

## Introduction

Repetitive transcranial magnetic stimulation (rTMS) is used to up- or down-regulate cortical plasticity and, a related application, to treat several neuropsychiatric diseases in humans such as chronic pain or depression (O’Reardon et al., 2007; Lefaucheur et al., 2014). Generally, high-frequency rTMS (≧ 5 Hz) is expected to increase and low-frequency rTMS (≦ 1 Hz) to decrease excitability although this claim strongly depends on intervals induced during stimulation (Rothkegel et al., 2010). Short-term modulation of cortical excitability that can induce longer lasting changes in synaptic plasticity is expected to play a key role in mediating TMS effects (Vlachos et al., 2012; Lenz et al., 2014; Noh et al., 2015; Trebbastoni et al., 2016). “Theta burst stimulation” (TBS; 3 pulses repeated at 50 Hz) protocols can induce changes in cortical plasticity in human subjects that outlast the stimulation period by up to 30–60 min (Huang et al., 2005). While several studies replicated and complemented these data (Di Lazzaro et al., 2005; Huang et al., 2007; Gamboa et al., 2010), the physiological responses to TBS protocols, like to other TMS or transcranial electric stimulation protocols, generally exhibit high intra- and interindividual variability (Maeda et al., 2000; Hamada et al., 2013).

Using TMS (Barker et al., 1985) it is possible to induce electric fields in the brain in a painless manner since, unlike with electric stimulation, the magnetic field passes easily through the skin and skull. In contrast, high-intensity electric stimulation is very painful because of the stimulation of skin receptors (Merton and Morton, 1980). Electric and magnetic stimulation protocols differ in the physical aspects of induced electric fields. While TMS primarily induces tangential currents, electric stimulation can create more varied field patterns depending on the electrode montage used. Also the restriction of essentially two pulse shapes, monophasic and biphasic, in conventional TMS devices can easily be overcome by the flexibility of electric stimulators. Thus the possibility of using electric stimulation offers more freedom for designing new stimulation protocols.

In general, electric stimulation protocols using weak intensities such as transcranial direct current (Nitsche and Paulus, 2000; tDCS) or alternating current (tACS; Antal et al., 2008) are gaining increased popularity in research and clinical applications due their easy implementation and flexibility.

It was shown in previous studies that transcranial alternating currents with frequencies in the lower kilohertz range could be safely applied with intensities of 1–2 mA (Turi et al., 2013; Chaieb et al., 2014). Interestingly, studies conducted directly at the dorsal root ganglions of the exposed spinal cords of rats showed that alternative current stimulation inhibits sensory neurons if applied in a frequency range of 2–100 kHz, thus potentially evoking an ameliorative effect on pain (Cuellar et al., 2013).

For this reason, we tried a novel method of applying TBS protocols using high-frequency, high-intensity electric currents. We applied electric stimulation in a TBS mode using 5 kHz high-frequency tACS. At frequencies below 1 kHz, tACS is believed to entrain or synchronize neuronal oscillations with effects on excitability but also behavioral aspects such as learning (Alekseichuk et al., 2016). If applied in the kHz range, tACS is thought to preferentially modulate the membrane excitability of neurons (Antal and Paulus, 2013). Continuous application of 2 kHz as well as 5 kHz tACS at 1 mA at the primary motor cortex (M1) led to a muscle evoked potential (MEP) amplitude increase, which outlasted the stimulation by an hour (Chaieb et al., 2011). While both the 2 kHz as well as the 5 kHz protocol used by Chaieb et al. (2011) showed a significant increase of MEP amplitudes, 5 kHz tACS produced a slightly larger effect, which is why we chose the 5 kHz protocol for the following study. Here we hypothesized that by using 5 kHz as a painless “carrier frequency” for electric pulses in a TBS pattern, we could achieve transcranial electric TBS effects through the use of an electrical theta burst stimulation (eTBS) protocol. We expected that a stimulation intensity of 10 mA in combination with the well investigated standard TBS pattern could compensate for the lower total stimulation time and the lower stimulation intensity of 1 mA used by Chaieb et al. (2011).

## Materials and Methods

### Subjects

Seventeen healthy subjects aged 24.4 ± 3.4 years (9 females/8 males) were recruited for the first part of the study, of which three were left-handed, and 14 healthy subjects aged 24.9 ± 4.5 years (8 females/6 males) were recruited for the second part of the study, of which none were left-handed. Two of the subjects in the first part of the study (1 female, 1 male) and four in the second part (3 female, 1 male) withdrew from the experiment due to painful skin sensations during eTBS. Since the second part of the study consisted of relatively high stimulation intensities, we introduced a sensation questionnaire which each participant had to complete after every session. None of the subjects had any metallic implants, history of neurological disease, were pregnant or took any medications at the time of the study. They all gave written informed consent and were compensated for participating. The investigation was approved by the Ethics Committee of the Medical Faculty of University of Göttingen, and conformed to the principals laid down in the Declaration of Helsinki.

### Transcranial Magnetic Stimulation

As previously described by our lab (Batsikadze et al., 2013; Sommer et al., 2013) a Magstim 200 magnetic stimulator (Magstim, Whiteland, Dyfed, UK) with a figure-of-eight magnet coil (diameter of one winding: 70 mm; peak magnetic field: 2.2 T) was used to create monophasic single-pulse TMS pulses to measure changes in corticospinal excitability. To induce MEPs, the coil was held tangentially to the scalp over the hand region of the motor cortex, with the handle pointing backwards and laterally at 45° from midline. The optimal position for the coil (hotspot) was established by inducing consistent 1 mV MEP amplitudes at the contralateral first dorsal interosseous (FDI) muscle. Resting motor threshold (RMT) was defined as the minimum stimulator output needed to elicit a MEP response of ±0.5 mV in the relaxed FDI muscle in at least 5 out of 10 consecutive trials. The MEPs were recorded using Ag-AgCl surface electrodes in a belly-tendon montage. The signals were amplified and band-passed filtered (2 Hz to 2 kHz, sampling rate 5 kHz, amplifier gain: 1000), digitized by a power 1401 AD converter (Cambridge Electronic Design, Cambridge, UK), controlled by Signal Software and stored for offline analysis. To ensure that the TMS coil was positioned at the same point during each measurement, a dermatologically tested tattoo marker was used to mark the hotspot.

### Experimental Design

The study was performed in a double-blinded, cross-over design. The eTBS protocols were encoded using Signal Software and applied in a randomized manner. The subject was seated in a comfortable chair with head and arm rests and was instructed to relax whenever necessary. For each subject, 25 MEPs were recorded per measurement time point. To establish a reliable baseline, two TMS measurements were done prior to the eTBS with a 10 min break in between. To evaluate stimulation after-effects, seven consecutive TMS measurements were performed following the eTBS; they started immediately after the stimulation and were made every 10 min for up to 60 min. Mean and SEM were calculated for each time point and compared to the baseline. The interval between the different sessions of each subject was set to at least 1 week. The experiments were performed either between 09:00–12:00 a.m. or 03:00–07:00 p.m.

### eTBS

**Figure 1:**
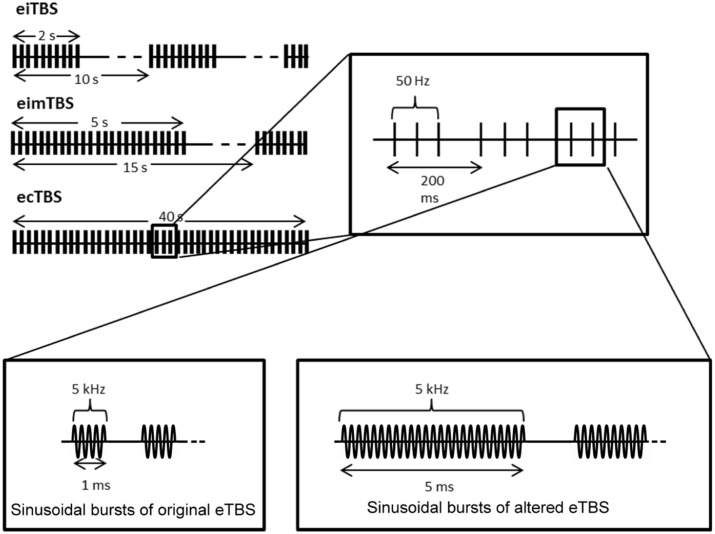
**Schematic illustration of the electrical theta burst stimulation (eTBS) protocols.** Each eTBS pattern consists of three pulses given at 50 Hz, repeated every 200 ms. Intermittent eTBS (eiTBS) uses a 2 s train of eTBS, repeated every 10 s. Intermediate eTBS (eimTBS) uses a 5 s train of eTBS, repeated every 15 s. Continuous eTBS (ecTBS) uses a 40 s train of uninterrupted eTBS. The first part of the study uses sinusoidal bursts with a length of 1 ms at 5 kHz, whereas in the second part of the study a burst length of 5 ms at 5 kHz was used in addition to the original sinusoidal bursts. The basic TBS pattern were obtained and modified from Huang et al. (2005).

eTBS was applied with one electrode (3 cm × 4 cm; cable facing posterior direction) placed over the left M1 (M1 region) and one electrode (6 cm × 8 cm; cable facing lateral direction) over the contralateral orbit at an intensity of 5 mA. The electrical current was provided by a DS5 isolated bipolar constant current stimulator (Digitimer, Welwyn Garden City, UK), which was run by a script using the Signal Software (Cambridge Electronic Design, v. 2.16). During the first part of the study, three eTBS patterns were applied as described by Huang et al., 2005: intermittent eTBS (eiTBS), intermediate eTBS (eimTBS) and continuous eTBS (ecTBS; Figure 1). Each eTBS pattern consisted of three stimulation pulses at 50 Hz, repeated every 200 ms for a total of 600 pulses each. Each of these three pulses consisted of five sinusoidal bursts at 5 kHz, repeated every millisecond. eiTBS uses a 2-s train of eTBS repeated every 10 s for a total of 190 s. eimTBS uses a 5-s train of eTBS, repeated every 15 s for a total of 110 s. The ecTBS pattern consists of a 40-s train of uninterrupted eTBS. An inactive sham stimulation was used as a control. To ensure optimal blinding, the duration of each eTBS script was set to 240 s, even though the actual stimulation duration of each protocol was shorter.

In the second part of the study, every subject received each of the following altered ecTBS protocols: ecTBS with sinusoidal bursts of 1 ms duration and 10 mA intensity, ecTBS with sinusoidal bursts of 5 ms duration and 5 mA intensity and ecTBS with sinusoidal bursts of 5 ms duration and 10 mA intensity.

### Computational Modeling of Current Distribution

A simulation of the electric field distribution in the brain for the electrode montage used in the experimental protocol (Figure 5A) was performed using SimNIBS^1^ (Windhoff et al., 2013; Thielscher et al., 2015) using the standard head model provided by the software. A current intensity of 5 mA was used in the simulations.

**Figure 2:**
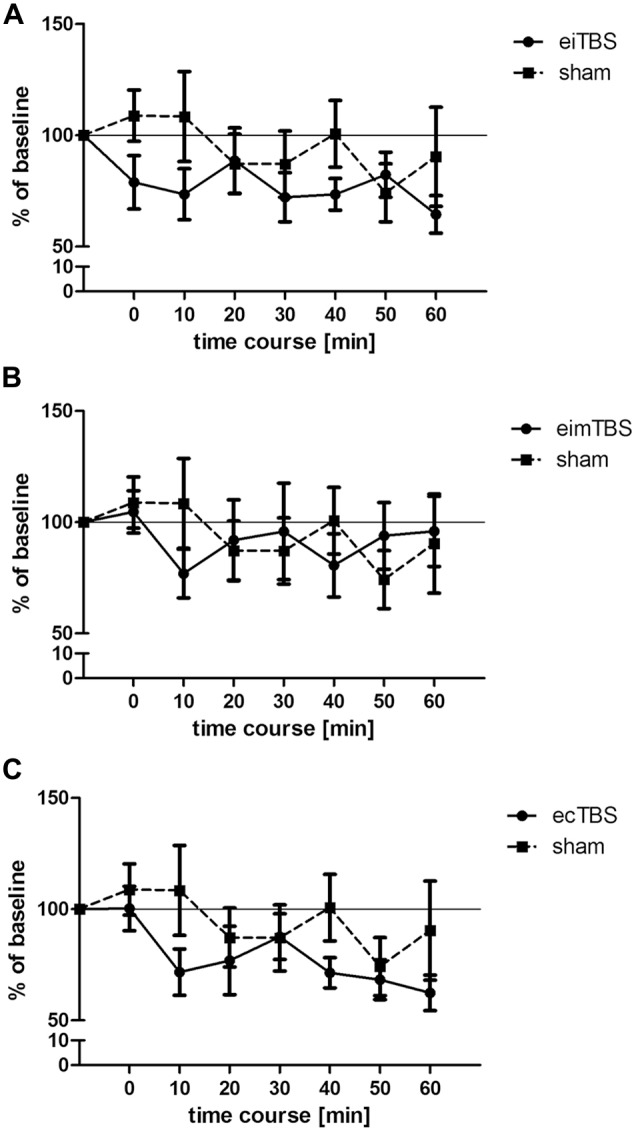
**Changes in muscle- evoked potential (MEP) amplitudes after eiTBS/eimTBS/ecTBS.** MEP size was measured at baseline and at 0, 10, 20, 30, 40, 50, 60 min after the stimulation. Data was normalized to baseline. Shown are mean values of 15 healthy subjects after eiTBS, eimTBS, ecTBS and sham stimulation, vertical bars denote standard errors. **(A)** eiTBS showed a significant inhibition of MEP size within the first 10 min (*p* < 0.05) after stimulation and again at the time points 30 (*p* < 0.05), 40 (*p* < 0.01) and 60 (*p* < 0.005) compared to baseline. **(B)** eimTBS showed no significant inhibition or facilitation of MEP size if compared to baseline or sham. **(C)** ecTBS showed a significant inhibition of MEP size at the time points 10 (*p* < 0.05), 20 (*p* < 0.05), 40 (*p* < 0.005), 50 (*p* < 0.01) and 60 (*p* < 0.005) compared to baseline. Compared to sham stimulation, ecTBS showed a significant inhibition of MEP size at the time point 10 (*p* < 0.05).

**Figure 3:**
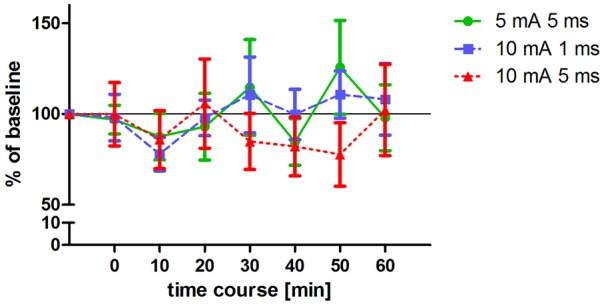
**Changes in MEP amplitudes after altered ecTBS.** MEP size was measured at baseline and at 0, 10, 20, 30, 40, 50, 60 min after the stimulation. Data was normalized to baseline. Shown are mean values of 10 healthy subjects after intervention with three altered ecTBS protocols, vertical bars denote standard errors. No significant effect on MEP amplitude compared to baseline could be observed.

**Figure 4:**
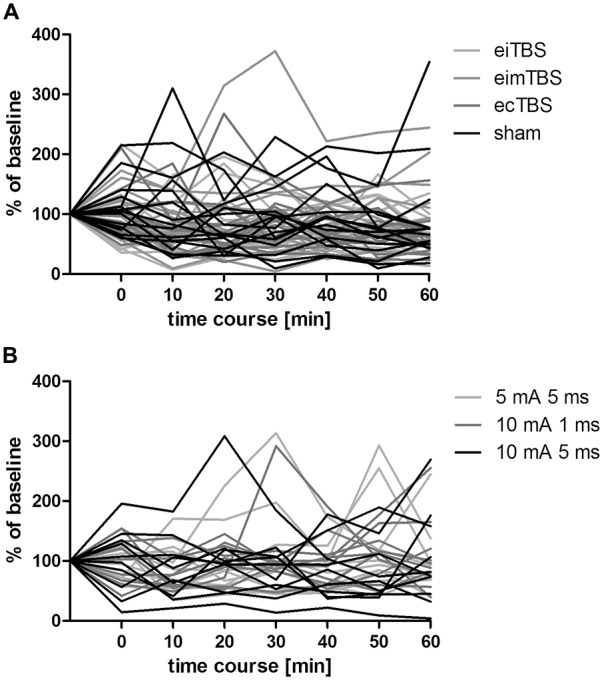
**Individual MEP responses to different eTBS protocols.** Individual MEP responses after **(A)** eTBS and **(B)** altered ecTBS of 15 and 10 subjects, respectively. Data was normalized to baseline.

**Figure 5:**
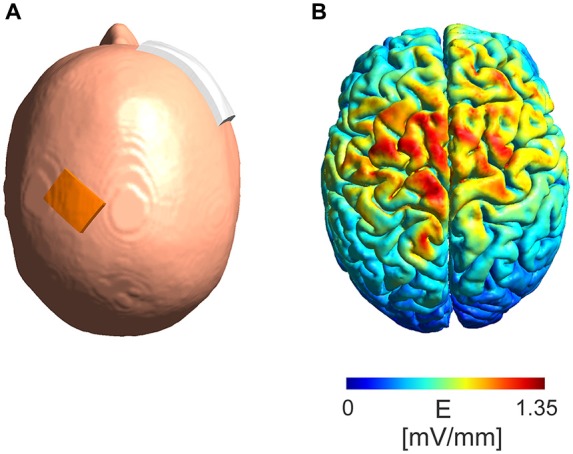
**Model of absolute electric field distribution. (A)** Depicted is the electrode montage with a 3 cm × 4 cm electrode placed over the M1 region and a 6 cm × 8 cm electrode placed over the contralateral orbit. **(B)** A simulation of the electric field distribution in the brain with a maximum of 1.35 mV/mm in the area between the electrodes.

### Data Analysis

Data were analyzed using GraphPad Prism for Windows version 5.0.0. MEP dataset was normalized to baseline, respectively. Due to the explorative nature of this study, a repeated measures ANOVA followed by Bonferroni’s Multiple Comparison Test was used on normalized data to compare each eTBS condition vs. sham. *T*-tests were performed on raw data to evaluate *p*-values for each time point compared to baseline.

## Results

### eTBS

Three different patterns of eTBS and one sham control stimulation were delivered to 15 healthy subjects on different days with a 1-week break between each session. MEP size was measured at baseline and at 0, 10, 20, 30, 40, 50 and 60 min after the stimulation. The average baseline MEP value was 1.01 ± 0.2 mV. Each dataset was normalized to the respective baseline. When all three eTBS protocols and the sham stimulation were compared, repeated measurements ANOVA revealed no significant main effect of TYPE of stimulation (*F*_(3,392)_ = 0.76; *p* > 0.05). Further repeated measurements ANOVA were performed for each stimulation protocol compared to sham. Neither eiTBS (*F*_(1,196)_ = 1.34; *p* > 0.05), nor eimTBS (*F*_(1,196)_ = 0.02; *p* > 0.05), or ecTBS (*F*_(1,196)_ = 1.3; *p* > 0.05) showed a significant effect of TYPE of stimulation if compared to sham. ecTBS showed a significant effect of TIME (*F*_(1,196)_ = 2.853; *p* < 0.05), due to the decrease of MEP sizes over the time course of 60 min. Compared to baseline, eiTBS showed a significant inhibition of MEP amplitude within the first 10 min (*p* < 0.05) after stimulation and again at the 30-min (*p* < 0.05), 40-min (*p* < 0.01) and 60-min (*p* < 0.005) time points (Figure 2A). No significant changes in MEP amplitude were however observed when compared to sham stimulation. eimTBS showed no significant effect on MEP sizes compared to baseline or to sham stimulation (Figure 2B). ecTBS showed a tendency similar to eiTBS towards an inhibitory effect on MEP size, with a significant inhibition compared to baseline at time points 10 (*p* < 0.05), 20 (*p* < 0.05), 40 (*p* < 0.005), 50 (*p* < 0.01) and 60 (*p* < 0.005). Here too, no significant effects could be observed compared to sham stimulation. The sham stimulation also showed no significant modulation of MEP amplitudes compared to baseline. Common to all three eTBS patterns is a decrease in MEP size 10 min after stimulation.

### Altered ecTBS

Three altered ecTBS protocols were used in the second part of the study: 5 mA ecTBS with sinusoidal bursts of 5 ms duration, 10 mA ecTBS with sinusoidal bursts of 1 ms and 10 mA ecTBS with sinusoidal bursts of 5 ms. Since we used higher stimulation intensities and longer sinusoidal bursts during this part, we introduced a sensation questionnaire which each participant had to complete after every session. Five out of the 10 remaining subjects reported slight to mild tingling sensations, while one participant reported a mediocre tingling sensation. Another participant reported a mildly uncomfortable sensation during the 5 mA ecTBS protocol with sinusoidal burst duration of 10 ms, which did not occur during the next stimulation sessions with 10 mA. Only one participant reported phosphenes during one of the ecTBS protocols (10 mA + 1 ms sinusoidal bursts). The average baseline MEP amplitude was 1.01 mV ± 0.21 mV. No significant effect could be observed on comparing the MEP amplitude of each stimulation protocol to the baseline (Figure 3). Figures 4A,B depict the individual MEP responses of each participant for both study parts. The MEP data is highly variable and no clear after-effect for any of the eTBS protocols could be demonstrated.

### Current Distribution

The modeled absolute electric field distribution (Figure 5B) for a 5 mA intensity shows a maximum electric field strength of 1.35 mV/mm in the area between the stimulation electrodes reaching parts of the frontal lobe at the ipsi- as well as contralateral hemisphere.

## Discussion

The translation of the theta-burst protocols from TMS which induces electric field strengths of about 100 mV/mm (Huang et al., 2005) to a similar patterned electric alternating current stimulation did not produce the anticipated effects on MEP amplitudes by eiTBS or ecTBS, respectively (Huang et al., 2005). TBS, as introduced by Huang et al. (2005), features a biphasic TMS pulse shape for which the second phase dominates the direction of the effect. eTBS as used here features sinusoidal bursts of 1 ms and 5 ms duration, and 5 mA and 10 mA intensities peak to baseline. Otherwise we employed the same TBS pattern as published by Huang et al. (2005). We used a 5 kHz carrier frequency in order to avoid or at least minimize skin pain as known from high pulse electric stimulation (Merton and Morton, 1980) and to reach higher field strengths.

The main reason why eTBS lacked the ability to modulate MEP responses is most likely the difference in intensities used during the magnetic and electric TBS. Magnetic TBS produces electric fields of 100 mV/mm for brief periods while eTBS produces sinusoidal waveforms with peak-to-baseline amplitudes of a maximum 10 mA at the scalp in this study. As demonstrated by our computational modeling, eTBS results in electric fields of up to 1.35 mV/mm for 5 mA in the cortical area after passing through the skull. Deans et al. (2007) showed that such electric fields are already sufficient to produce subtle changes in transmembrane potentials and thus increase the probability of action potential firing when applied in a low frequency AC fashion. These changes in transmembrane potentials show a trend to saturation at a frequency of 100 Hz, although no investigations were performed in the kHz frequency range (Deans et al., 2007). The present study is based on the findings that the application of low kHz tACS interferes with membrane excitability, leading to a modulation of cortical plasticity (Chaieb et al., 2011). While there is only a minor effect on transmembrane potentials with electric fields of low intensity such as 1.35 mv/mm, we expect a summation of this effect to lead to significant changes in nerve excitability. It is thought that human pyramidal neurons can regulate time action potential firing with sub-millisecond precision (Testa-Silva et al., 2014). Nevertheless, the frequency of 5 kHz as used in the present study could prove to be too fast to interfere with spike timing when applied only for 5 ms. An increase of the spike duration from 5 ms to, as an example, 10 ms could proof to be more efficient in modulating the membrane excitability by increasing the time by which the membrane is exposed to changes in the voltage gradient. On the other hand, according to *in vitro* experiments performed by Stern et al. (2015) axons of rat neurons cultured at room temperature and stimulated with global extracellular electric fields show a chronaxie of 100 μs. Taking into account that human cortical neurons can track high frequency inputs better than rodent cells (Testa-Silva et al., 2014), this implies that membranes of human cortical neurons should possibly be able to respond to frequencies of up to 10 kHz.

According to the summation principle described by Gildemeister (1943), AC stimulation in the kHz range, like the 5 kHz frequency of eTBS, may be able to decrease the threshold voltage for nerve excitation. We assumed that this reduction in threshold voltage would lead to a modulation of MEP response after eTBS at the M1. However, no such modulation could be observed when compared with placebo stimulation. Interestingly, we found a significant reduction in MEP response with the lower stimulation intensities (Figure 2), if compared to baseline, but not with the higher intensities (Figure 3), irrespective of the TBS pattern. It could be argued that the intensity of the electric field was not yet high enough or the stimulation duration not long enough to produce changes in the transmembrane potential that would give us the hypothesized summation effect as described by Gildemeister and as seen by Chaieb et al. (2011).

The electrode montage used in this study correlates with the montage commonly used for stimulation of the M1 region in several neurophysiological studies (Chaieb et al., 2011, 2014; Wach et al., 2013). Nevertheless, while a part of the electric field focused over the targeted hand area of the M1, the electric field spread further towards the contralateral electrode and covered part of the ipsi- and contralateral supplementary motor cortex. To increase the focality of the electric field over the M1 region one possibility would be to use the high definition electrode montage as shown by Datta et al. (2009) and Alekseichuk et al. (2016). It is known from other studies that when increasing stimulation intensities from a subthreshold level, at first inhibition occurs and then eventually, after passing a transition zone without a stimulation effect, excitation is induced (Moliadze et al., 2012). Although it is very speculative, it may be that the positive inhibitory findings in Figure 2 reflect a low intensity of stimulation in which the pattern of TBS does not play a role and the negative findings of Figure 3 reflect the transition zone.

We assume that the same motor cortex area in the sulcus of M1 (Laakso et al., 2014) is being activated here as in other studies using tACS of the motor cortex with a comparable electrode montage (Moliadze et al., 2010; Chaieb et al., 2011). When applying a posterior-to-anterior (PA)-directed current via TMS, it first activates the cortical layer 1 ending in layer 6, thus depolarizing the soma of pyramidal tract cells (Jefferys, 1981). An anterior-to-posterior (AP)-directed current would probably first activate the cortical layer 6 and finally layer 1, resulting in a soma-hyperpolarizing and dendrite-depolarizing effect on the pyramidal tract cells (Jefferys, 1981). AP-directed current has been associated with the facilitation of back propagating potentials (Sommer et al., 2013). This model explains why a PA pulse would need lower threshold intensity for inducing an action potential as compared to an AP pulse. The AC flow as used here would, by nature, interact with both PA- as well as AP-oriented neuronal structures and lacks direction specificity. This could be one possible explanation for our negative results using eTBS protocols. To circumvent this problem and to create a higher directionality, a tDCS offset could be used in combination with the eTBS protocols.

We made an effort to standardize our protocol by including only healthy subjects within a certain age range (18–45 years) and an equal number of female/male volunteers. Furthermore, we ensured that the experiments were always conducted between 09:00 am and 06:00 pm on each experiment day, using the same laboratory, and we standardized the stimulation duration for each eTBS protocol to allow for optimal blinding conditions. However many factors contribute to negative inter- and intraindividual results in cortical excitability (Maeda et al., 2000; Hamada et al., 2013). These include genetic differences like the brain derived neurotrophic factor (BDNF) polymorphism Val66Met, the awareness level of the subject or even the time of day (Ridding and Ziemann, 2010). Furthermore, it has been observed that a pre-activation of the target cortex area by motor activities or stimulation protocols such as tDCS, TBS or quadripulse stimulation (QPS) can also modulate MEP facilitation or inhibition (Lang et al., 2004; Ridding and Ziemann, 2010).

In summary, we demonstrated a method with which to apply high frequency tACS with intensities of up to 10 mA in a safe manner, although the given protocols were insufficient to produce lasting changes in cortical plasticity. Since electrical stimulation protocols like tACS or eTBS use different underlying mechanisms as magnetic stimulation protocols, one has to be cautious when comparing those stimulation types. More studies are needed to evaluate the potential of high-frequency stimulation of the human cortex in order to generate plastic after-effects.

## Author Contributions

PK conducted the experiments, analyzed the data and wrote the manuscript; AA analyzed the data and revised the manuscript; MH designed the experimental set up; AN analyzed the data and revised the manuscript; AO analyzed the data and wrote the manuscript; WP wrote and revised the manuscript and designed its content.

## Conflict of Interest Statement

The authors declare that the research was conducted in the absence of any commercial or financial relationships that could be construed as a potential conflict of interest.

## Footnotes

^1^www.simnibs.de

## Abbreviations

BDNF: brain derived neurotrophic factor
ecTBS: continuous eTBS
eimTBS: intermediate eTBS
eiTBS: intermittent eTBS
eTBS: electrical theta burst stimulation
FDI: first dorsal interosseous muscle
MEP: muscle evoked potential
RMT: resting motor threshold
rTMS: repetitive transcranial magnet stimulation
tACS: transcranial alternating current stimulation
tDCS: transcranial direct current stimulation
tES: transcranial electrical stimulation
QPS: quadripulse stimulation.
